# A two for one special: EEG hyperscanning using a single-person EEG recording setup

**DOI:** 10.1016/j.mex.2023.102019

**Published:** 2023-02-03

**Authors:** Caitriona L. Douglas, Antoine Tremblay, Aaron J. Newman

**Affiliations:** Dalhousie University, Halifax, Nova Scotia, Canada B3H 4R2

**Keywords:** EEG hyperscanning, Multi-modal psycholinguistic methods, Human interaction research, Event-related potentials, EEG hyperscanning using existing single-person EEG recording setup

## Abstract

EEG hyperscanning refers to recording electroencephalographic (EEG) data from multiple participants simultaneously. Many hyperscanning experimental designs seek to mimic naturalistic behavior, relying on unpredictable participant-generated stimuli. The majority of this research has focused on neural oscillatory activity that is quantified over hundreds of milliseconds or more. This contrasts with traditional event-related potential (ERP) research in which analysis focuses on transient responses, often only tens of milliseconds in duration. Deriving ERPs requires precise time-locking between stimuli and EEG recordings, and thus typically relies on pre-set stimuli that are presented to participants by a system that controls stimulus timing and synchronization with an EEG system. EEG hyperscanning methods typically use separate EEG amplifiers for each participant, increasing cost and complexity — including challenges in synchronizing data between systems. Here, we describe a method that allows for simultaneous acquisition of EEG data from a pair of participants engaged in conversation, using a single EEG system with simultaneous audio data collection that is synchronized with the EEG recording. This allows for the post-hoc insertion of trigger codes so that it is possible to analyze ERPs time-locked to specific events. We further demonstrate methods for deriving ERPs elicited by another person's spontaneous speech, using this setup.

• EEG hyperscanning method using a single EEG amplifier
• EEG hyperscanning method allowing simultaneous recording of audio data directly into the EEG data file for perfect synchronization
• EEG method for naturalistic language and human interaction studies that allows the study of event-related potentials time-locked to spontaneous speech

## Graphical abstract

**Figure:**
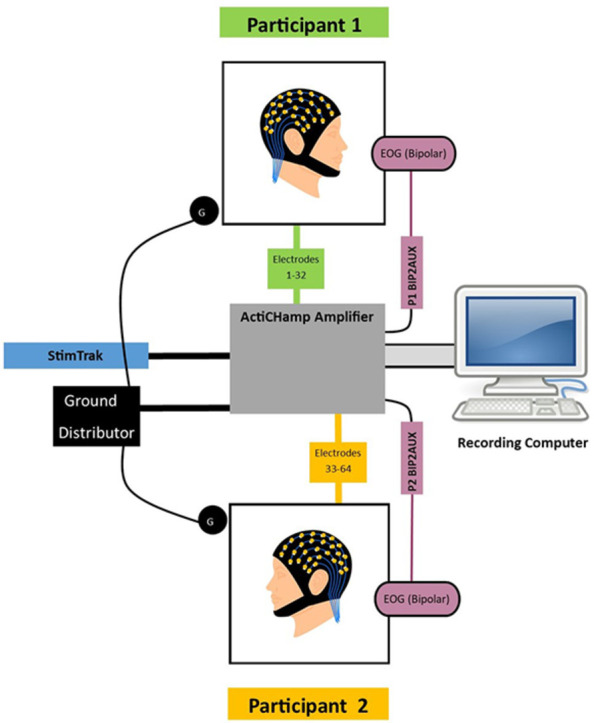

Specifications table

**Table:** 

Subject Area:	Psychology
More specific subject area:	*Electrophysiology*
Method name:	*EEG hyperscanning using existing single-person EEG recording setup*
Name and reference of original method:	Babiloni, F., Cincotti, F., Mattia, D., Mattiocco, M., De Vico Fallani, F., Tocci, A., Bianchi, L. , Marciani, M. G., & Astolfi, L.. (2006). Hypermethods for EEG hyperscanning. *2006 International Conference of the IEEE Engineering in Medicine and Biology Society,* 3666–3669. https://doi.org/10.1109/iembs.2006.260754
Resource availability:	Jupyter Notebook code scripts and the dataset mentioned in the paper have been provided to download alongside this paper in the link below: Free conversation: http://hdl.handle.net/10222/80821 Scripted conversation: https://osf.io/w2dc6/

## Method details

### Overview

EEG hyperscanning refers to recording electroencephalographic (EEG) data from multiple participants simultaneously, typically as they are jointly engaged in a particular task [1]. In EEG recording, electrodes placed on the head measure electrical potential at the scalp via a differential amplifier [2,3]. Current methods in EEG hyperscanning [4,5,6] typically involve using separate amplifiers for each person in the study. This creates challenges in synchronizing data between amplifiers. It also presents barriers to entry into conducting hyperscanning research, as the cost of amplifiers means that only labs that are equipped with multiple EEG systems can conduct this research.

Another, distinct challenge in conducting hyperscanning research is in measuring event-related potentials (ERPs). ERPs are averages of EEG activity time-locked to the onset of events of interest, such as the presentation of experimental stimuli [2]. Measuring ERPs requires precise time-locking of the EEG to events of interest, because ERPs show distinct peaks – with associated functional interpretations — at precise times. Thus, variance of even tens of milliseconds in the synchronization of EEG and stimuli can result in uninterpretable data. ERP studies typically employ pre-set stimuli that are presented by a system that sends a “trigger code” time-locked to each stimulus, to the EEG recording system. In a properly calibrated system, this trigger code acts as a time stamp in the EEG data to which ERPs can be reliably derived [2,5]. In hyperscanning studies, experimental designs typically seek to mimic naturalistic behavior and thus rely on unpredictable, participant-generated stimuli. For this reason, the majority of EEG hyperscanning studies so far have not focused on ERPs but rather on neural oscillatory activity [6]. This type of rhythmic activity is measured as power in specific frequency bands, averaged over periods of hundreds of milliseconds or even seconds. While oscillatory activity is one important way of understanding human brain activity, ERPs can provide important additional insights into neurocognitive activity.

Designing experiments and recording data of a conversation or speech production task between multiple participants using an EEG hyperscanning approach presents a particular challenge as both a continuous time-series of behavioral data (e.g., audio, video) must be recorded alongside and synchronized with multiple EEG recordings. A few studies have demonstrated that this approach is feasible by using approaches that employed software to synchronize the EEG and audio and/or video data streams. For example, Fachner and colleagues [7] used a single EEG amplifier and 64 EEG electrodes that were split between two participants to record a music therapy session, while video (with audio) of the therapeutic interaction was recorded and synchronized with the EEG data using software that synchronized the video and EEG recordings during the recording process and then later analyzed the recordings to determine the timing of events for EEG analysis. Peréz and colleagues used trigger codes sent to the EEG system to mark the start and end of audio recordings and synchronized the audio and EEG recordings post-hoc [8]. Both studies recorded a continuous timestream of conversational interactions and synchronized these with EEG recording using software.

Because EEG and audio can both be recorded digitally as time-varying signals, it is also possible to record audio directly into an EEG data file, rather than relying on software synchronization post-hoc. In principle, this should provide the most reliable synchroniization between audio and EEG. To our knowledge, the hardware approach of joint EEG-audio signal recording has only been used in single-person and EEG hyperscanning to studies to verify and ensure the exact synchronization of the EEG signal and audio stimulus presented to the participants, not to record a real-time conversation between participants and synchronize the resulting audio data with the EEG recording [9,10,11]. Here, we present an EEG hyperscanning technique that uses a single EEG amplifier and an audio microphone accessory to record the EEG hyperscanning data of two participants and the audio data of a conversational interaction using this hardware level synchronization approach.

**Fig. 1:**
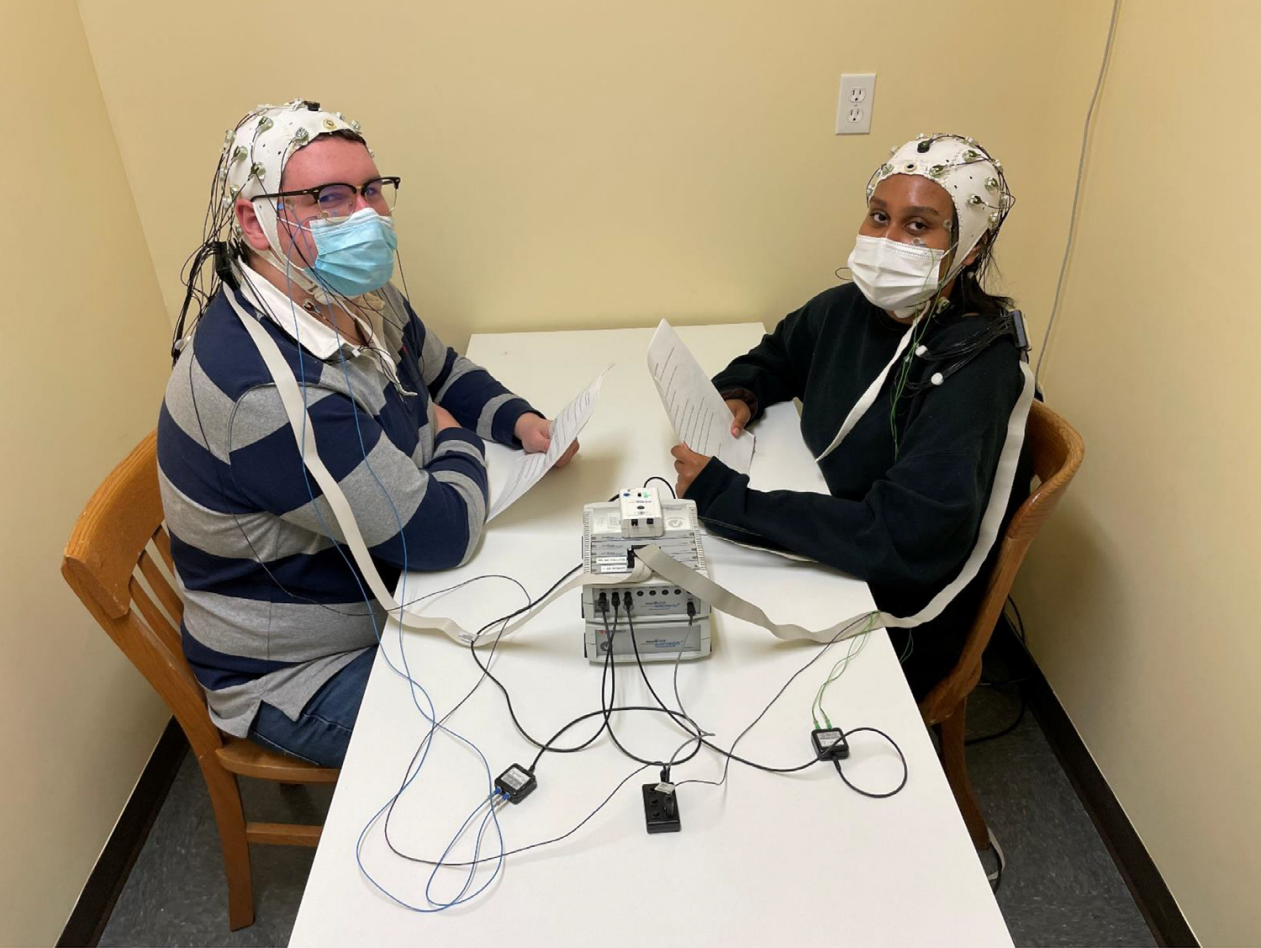
An image of two participants using the EEG hyperscanning setup. The amplifier and battery are in the center of the image, between the participants. On top of the amplifier is the StimTrak device. Below the amplifier in the image (close to the camera) is the ground distributor (center) and the BIP2AUX boxes used to record bipolar EOG from each participant. Note: Typically, the StimTrak device that is pictured on top of the EEG amplifiers is placed on the table centered between the two participants as depicted in Fig. 2. It was placed on top of the amplifiers here, so that it is in full view.

We have developed an EEG hyperscanning technique that could be used to investigate ERPs in response to words spoken in the context of conversation between two people, while also accommodating oscillatory analyses. As shown in Fig. 1, we employ a 2 × 32-electrode EEG hyperscanning setup that both allows for the simultaneous acquisition of EEG data from a pair of participants using a single amplifier. The setup also allows for simultaneous collection of audio data into an additional channel in the EEG recording. This ensures that the audio and EEG data are perfectly synchronized. Leveraging this, we additionally developed a method for manually identifying the onsets of individual words in the audio recordings and using these to insert trigger codes so that it is possible to derive and analyze ERPs time-locked to the onset of individual spoken words.

This EEG hyperscanning setup involves commercially-available equipment. As described in detail below, we have used a Brain Products actiCHamp EEG amplifier configured with 64 EEG channels. This uses active electrodes (ActiCap, Brain Products) which are provided in bundles of 32 electrodes. This makes it easy to apply 32 electrodes to the head of each participant. It should be noted that in a review of using different EEG hardware for hypersacnning studies, Barraza and colleagues [4] noted that, “Brain Products setup using actiCHamp is not described since this amplifier was not explicitly designed for EEG hyperscanning” (p. 346), noting that by default all channels share the same ground, so that noise specific to each individual participant cannot be isolated and removed. However, these authors also noted that, “…dual recordings have been successfully conducted with actiCHamp amplifiers by using a y-cable (GND distributor) that should be connected from the two GNDs coming from both participants into the GND port on the actiCHamp.” (p. 436). Indeed, this is the same approach used by Fachner and colleagues [7] and was also recommended to us by Brain Vision LLC, with the caveat that the approach “…seems to work in cases in which the noise levels between the two participants are pretty much equal and assuming that none of the two participants has more signal drift than the other” (F. Strelzyk, personal communication, Nov. 7, 2016). Likewise, actiCHamp amplifiers do not have a dedicated hardware reference electrode (let alone the ability to place a separate reference on each participant), and instead by default use reference-free recording (in which each channel represents the difference between one electrode and the ground), with the option of adding a reference electrode in software during recording, or post hoc during preprocessing. As noted in the actiCHamp amplifier manual [12], with reference-free recording “The common mode rejection against identical artifacts in all signals is weaker than in referential recording mode. In this mode the line noise artifacts are proportional to the electrode impedances.” (p. 65). In the present approach we used reference-free recording combined with the ground distributor, and then offline we separated the data by participant, and re-referenced each participant's data to the average of all electrodes that had been placed on their body only. In this way, we applied common-mode rejection offline by subtracting any variance common to all electrodes on each individual from their data. In principle, if the noise levels or amount of drift were different between the two individuals during recording, this approach could still result in noisier recordings than had we used dedicated amplifiers, each with their own ground and hardware reference electrodes, for each participant. However, as we demonstrate below, in practice the data had sufficient signal-to-nosie ratio to reliably detect the ERP component of interest in this study.

Another critical component of our setup is the Brain Products StimTrak device. This device allows us to record the audio data as an auxiliary EEG channel, which ensures that the audio recording of the conversational events is synchronized to the EEG data. Below we describe in detail the setup, procedures, and analysis pipeline we have developed, and demonstrate its validity using data obtained from two people engaged in a conversation. We hope that the description of a novel hyperscanning setup that uses a single EEG amplifier and that collects audio data as an accessory EEG channel that allows for the study of both neural oscillatory and ERP analyses will allow for EEG hyperscanning research to become a more common practice.

### Materials

EEG system and accessories:

• 64-electrode silver-silver chloride coated active ActiCap active electrodes (Brain Products, Gilching, Germany), provided as two bundles of 32 electrodes each
• 4 silver-silver chloride disk electrodes (for electrooculogram — EOG — monitoring)
• Brain Products actiCHamp 64 channel EEG amplifier (Brain Products)
• StimTrak device (Brain Products)
• ActiCap ground distributor and two ground electrodes (Brain Products)
• 2 BIP2AUX adapters (Brain Products)
• 2 flexible fabric EEG caps

EEG consumables:

• Electrolyte gel compatible with the EEG system (e.g., Brain Products’ SuperVisc)
• Exfoliating skin prep gel (e.g., Nuprep; Weaver and Company, Aurora, USA)
• Double-sided circular medical adhesives for electrodes
• Plastic electrode washers

Supporting Technology Requirements:

• Computer running Windows operating system, for recording EEG, with the following software installed:
  ○ PyCorder recording software (Brain Products)
• Computer for data analysis, with the following software installed:
  ○ Praat version 6.1.42 [13]
  ○ Python version 3.6 or higher
  ○ MNE-Python package and associated dependencies (see https://mne.tools/) [14]

### EEG equipment configuration

**Fig. 2:**
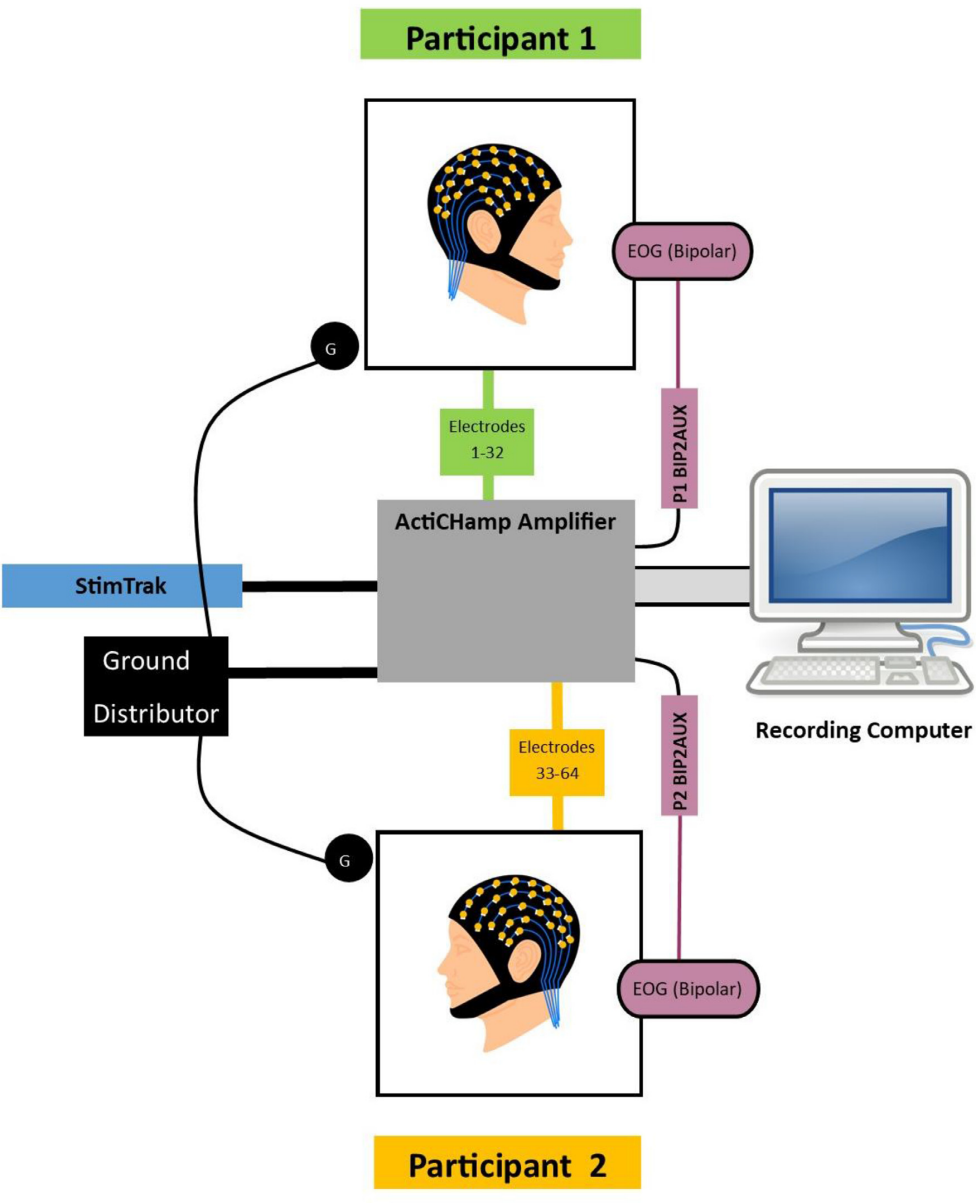
A diagram of the EEG hyperscanning setup. This uses a single, 64-channel EEG amplifier which takes as input two separate bundles of 32 electrodes each. For hyperscanning, one bundle of 32 electrodes is placed on each participant's head. Ground electrodes placed on the scalp of each participant are connected to a ground distributor device (Brain Products) that connects to the ground input on the amplifier. EOG is captured using a bipolar pair of electrodes placed around each participant's eyes, which connect to AUX ports on the amplifier via Brain Products BIP2AUX devices. Audio is recorded from both participants using the microphone built into the StimTrak device (Brain Products), which is connected to the EEG amplifier. As a result, EEG from two participants, as well as audio during the study, are recorded synchronously through a single amplifier and recording computer, to a single data file.

Fig. 2 shows the configuration of EEG recording equipment. Details of the setup and procedure are below.

### EEG recording settings

Brain Vision's PyCorder software is used to record the EEG and audio data. PyCorder requires the user to set up a recording configuration prior to data collection. It is important to set up the configuration so that each recording channel is clearly labelled according to the participant that the channel is recorded from. Thus, we manually labelled channels 1–32, as well as the first bipolar EOG channel, with the prefix “P1_” and channels 33–64 as well as the second bipolar channel with the prefix “P2_”.

The audio of the conversation is recorded as an accessory EEG channel using the Stimtrak device (Brain Products). While EEG data for cognitive ERP or oscillatory analyses typically do not need to be sampled at more than 250 Hz, speech needs to be sampled at a much higher rate in order to be intelligible. We therefore set the sampling rate to the maximum possible on our system, 10 000 Hz. We found that the quality of the recorded audio was sufficient to identify each word that was spoken, and to identify its onset with sufficient precision. We have provided a copy of the audio recording from the scripted conversation example dataset as supplementary material.

### EEG electrode application

First, we measured the participants’ heads and determined their EEG cap size. We prepared the faces of the participants by applying an exfoliating skin prep gel (Nuprep) on the areas where electrodes would be placed, including the periorbital region (lateral to left eyebrow and below the left eye), the left and right masseter regions, and on either side of the larynx. From here on, the electrodes on the masseters and larynx will be referred to as EMG electrodes as we used them to monitor facial muscle activation. We used double-sided circle shaped medical adhesives to attach to the bipolar electrodes and the EMG electrodes washer.

**Fig. 3:**
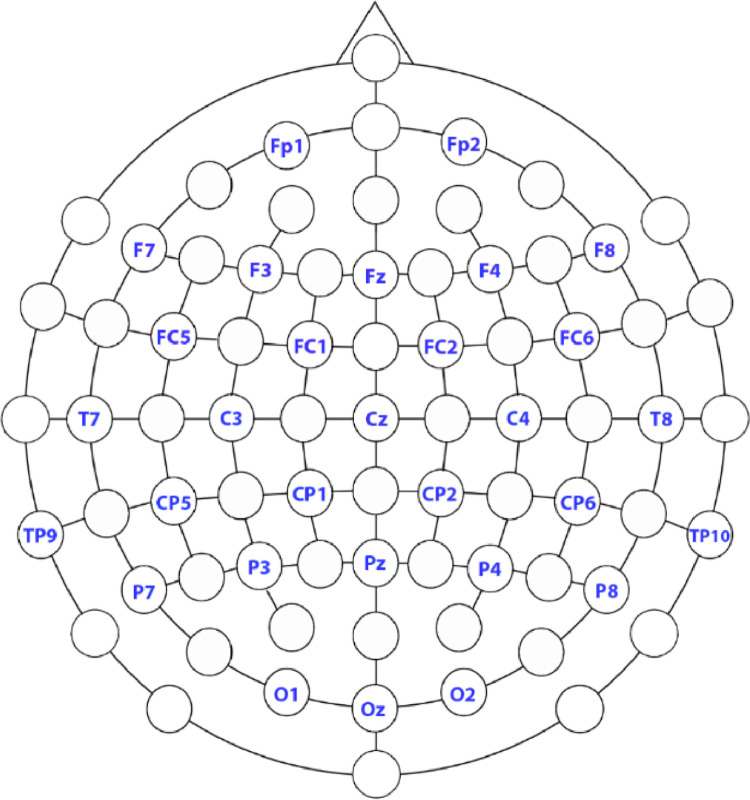
The 28-electrode EEG electrode recording montage for a single participant, with positions based on the International 10–10 System. The system used is a 32-electrode system, but 4 electrodes are used as EMG electrodes (see Fig. 4).

**Fig. 4:**
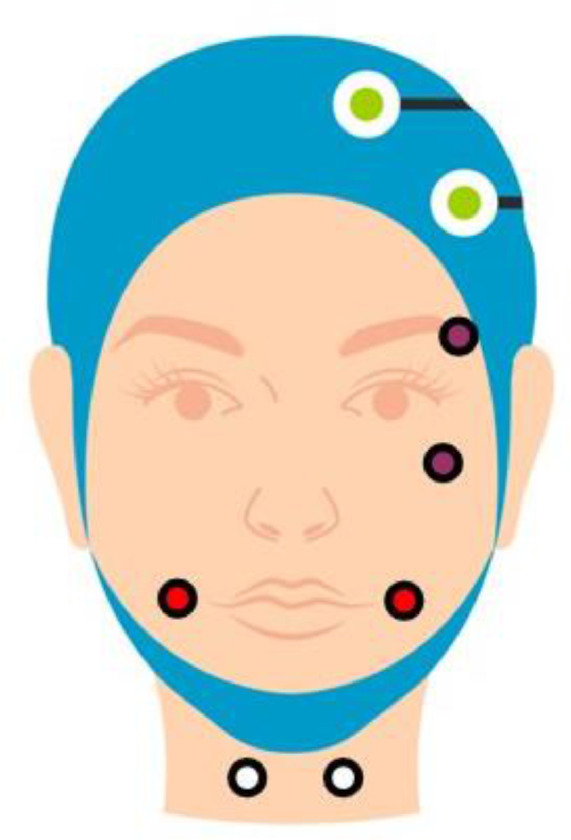
Illustration of placement of the EOG and EMG electrodes. The two electrodes shown in purple by the eye are the EOG electrodes, which are recorded as a bipolar pair. The electrodes shown in red and white were positioned to monitor EMG activity associated with speaking. The electrodes on either side on the mouth, shown in red, were placed over masseters, and the electrodes shown in white were placed on either side of the larynx.

When putting the EEG cap on each participant, the chin strap was tightened to ensure it remained stable and snug fitting. After putting on the EEG cap, we attached a ground electrode to each cap and connected these to the ground distributor. The ground distributor was plugged into the ground connector on the EEG system. Then, the EMG electrodes were applied to the participants. Electrodes 1 and 2 from each 32-electrode bundle went on the face of one participant, and electrodes 17 and 18 from each bundle were placed on either side of the larynx (see Fig. 3 for illustration of placement). EMG data was recorded from each electrode referenced the same as scalp electrodes, and then to bipolar measurements during postprocessing (see below). The remaining 28 electrodes of each bundle were attached to the electrode cap according to the recording montage depicted in Fig. 3. Participant 1 was equipped with electrode bundle 1–32 (see Fig. 4) and Participant 2 was equipped with electrode bundle 33–64 (see Fig. 4).

After the electrodes are placed on the participant, electrolyte gel was placed in the gap between the electrode and scalp using a blunt-tipped syringe, and then we gently rubbed the gel under the electrode with the syringe tip to ensure that there was a good connection between the scalp and the electrode. Following the setup of the electrode array, the electrodes were plugged into the amplifier, which was in turn connected to the computer responsible for recording the data using PyCorder. In PyCorder, we loaded the recording montage. We performed an impedance check using PyCorder to verify that all electrodes had a low impedance level (less than 25 kOhm, following manufacturer's guidance given the input impedance of the amplifier). We found that the impedance of the ground electrodes on both participants should be lowered as much as possible first, before checking and lowering impedances at other electrodes. This is because impedance is measured relative to the ground input on the amplifier. Since the two ground electrodes connect to the single ground input on the amplifier, the impedance of the ground electrodes on both heads affects impedance measurements at other electrodes on either head. If any electrodes had a high impedance, we rubbed the area under the electrode gently, and added small additional amounts of electrolyte gel as needed, until the impedance was below 25 kOhm.

### Data acquisition

First, the StimTrak device was configured and then turned on. The Trigger Level setting should be set at 1 V and the Gain dial should be set to “Mic”, otherwise the audio recordings are not intelligible. The Signal Out port on the StimTrak should be connected to the AUX 1 port on the actiChamp amplifier. When the StimTrak was connected to other auxiliary ports, it was not detected in PyCorder. We found that while the StimTrak has a low battery light, it often failed to appear in PyCorder, so we used freshly charged batteries in the StimTrak for each participant pair to ensure that it did not fail during recording. To check for audio recording, we viewed the auxiliary audio channel in PyCorder and ensured that the sampling frequency on display was set at 10 000 Hz. Then we spoke and clapped to confirm that the audio waveforms of these occurrences were displayed in PyCorder.

Finally, we started recording the EEG data using PyCorder. Participants performed the experimental task, which involved having a conversation. To date in our lab, we have applied this method to three paradigms: (1) a scripted conversation (which allows us to manipulate linguistic features of the words used, and derive averages for the same linguistic items across participants); (2) having two participants watch a pre-recorded, scripted conversation, and (3) having two people engage in a free (unscripted) conversation. The two datasets provided alongside this paper as supplementary material are from the free conversation and the scripted conversation experiments. For these experiments we were interested in the listening participant's neural response to the words produced by the speakers. Participants take conversational turns acting as speaker and listener. When the task was complete, we stopped the EEG recording and removed the electrodes and gel from participants.

### Data processing

The supplementary materials include two sample datasets that contain the data participant pairs engaged in conversation, a spreadsheet file of word onset timings, and two sample Python scripts (in Jupyter notebook format) detailing the processing steps using the MNE-Python package [14]. The scripted conversation dataset is hosted on the Open Science Framework (https://dx.doi.org/10.17605/OSF.IO/W2DC6) and the step 1 script can be used to generate an audio recording. The free conversation dataset is hosted on the Dalhousie Library repository (http://hdl.handle.net/10222/80821). Please note that the original audio of the free conversation dataset cannot be provided due to ethical constraints, as the participants’ data must remain un-identifiable, so the audio channel has been removed from the sample dataset. The scripts are meant to function as both tutorial and validation of the methods. In the first script (labelled Step_1), we describe how to import a raw EEG data file from a dyadic recording as described above, extract the audio recording and export it to an audio file (WAV format) using the *wavfile.write()* function in the scipy Python library, concatenate the raw data, remove the accessory channels so that only the EEG channels of interest remain, and decimate the data to 500 Hz for ease of future data analysis (improving computational speed and reducing data storage demands). Running the first script (Step_1) will extract the audio file from the EEG recording of the scripted conversation task provided as supplementary data. The second script (labelled Step_2) was used to perform data preprocessing and ERP segmentation once word onset timings were derived from the audio file. A text file (in CSV format) has been provided with a transcript of the words and their onset times that were used as events alongside the dataset and the sample Python scripts.

For our study, we identified the onsets of nouns that participants spoke during their conversation by loading the audio file of the conversation into the Praat software package [13]. By listening to the recordings while viewing the waveforms of the audio, trained coders identified the onset timing of each word of interest (in our example data, each noun) and entered these in a spreadsheet. The template spreadsheet contained a row for each word of interest, including columns for the identity of the word, which participant it was spoken by, the onset timing of the word (relative to the start of the audio file, which was also the start of the EEG recording), and a column that allowed coders to note if the word should not be used in analysis, for example if it was misspoken, a repetition of previous utterance, or otherwise unintelligible.

The spreadsheet was then imported into the second MNE-Python data processing script (labelled Step_2) and used to generate event codes. Because the audio file was extracted from the original EEG data recording, the timings identified in Praat are directly usable as timings for event code markers for ERP extraction. In MNE-Python the event codes can be stored in 3-column array with the first column containing the timings of the events, the second column being zeros (unused), and the third being a numeric code identifying the type of event. For present purposes, in this column we simply coded trials with a 1 if they were heard by Participant 1 (i.e., spoken by participant 2) and a 2 if they were heard by Participant 2.

In addition to the post-hoc trigger code insertion process, the second MNE-Python script demonstrates our entire EEG preprocessing pipeline through to deriving ERPs for each word. The second script also demonstrates how we performed preprocessing on each individual recording. In general, these follow established procedures [2,^3^,15], and no unique preprocessing steps were required other than separating the dyadic recording into two individual data sets as described below.

Bandpass filtering was performed on the continuous EEG data prior to separation into two individuals’ data sets. Two filtered copies of the data were produced for each participant: both used a lowpass cutoff of 20 Hz, while one used a highpass cutoff of 1.0 Hz (for input to ICA; see below) and the other a highpass cutoff of 0.1 Hz (to which the ICA corrections were applied, and which was used for subsequent analysis).

The filtered, continuous EEG data were then divided into separate data sets for each individual, by removing the channels associated with the other participant, and re-referencing to the average of only the electrodes placed on the scalp of that participant (which removed a significant amount of noise associated with the shared ground during recording). Subsequent processing steps were applied to each participant's data separately. The continuous data were segmented into ERP epochs each running from 200 ms prior to the onset of each word to 1000 ms after. Each epoch was baseline-corrected by subtracting the mean of the 200 ms preceding event onset from the entire epoch, and also removing the linear trend. We found this latter step improved data quality by reducing low-frequency drift that remained after filtering. Epochs were then visually inspected, and we manually identified and removed any excessively noisy trials. We then decomposed the data using independent components analysis (ICA; using the *fastica* algorithm [16]), and identified and removed components deemed to be dominated by ocular or EMG artifacts [17]. Subsequent to ICA artifact correction, data for each trial were baseline corrected by subtracting the mean amplitude calculated from the 200 ms period preceding the onset of each word, and data from any scalp channels removed earlier (due to bad electrodes) was interpolated.

### Proof of concept demonstration

**Fig. 5:**
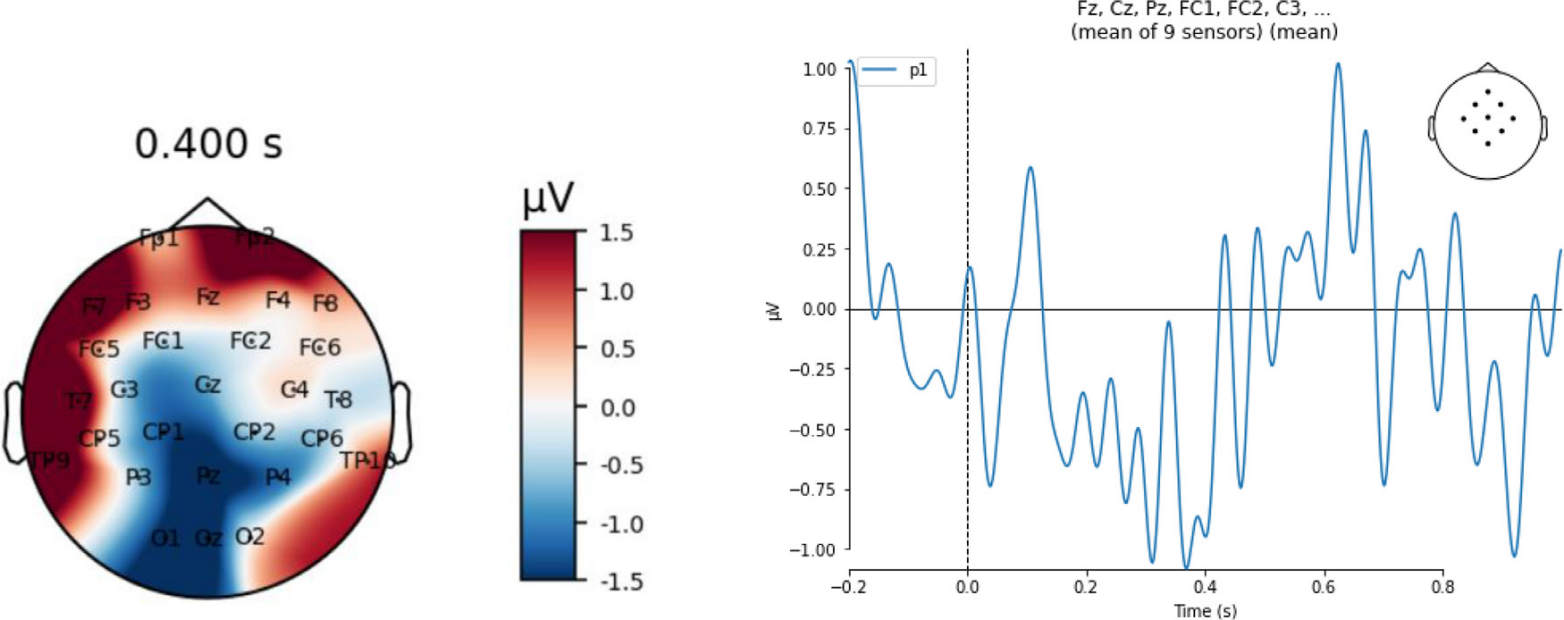
Data from one participant participating in a free conversation, averaged across all nouns heard by that participant. The left panel shows a scalp topography map of activity averaged over 300–500 ms. The right panel shows anwaveform plot of the average across a group of central-parietal electrodes (shown in the top right inset), time-locked to word onset (time 0). Both display a negative potential over the midline central-parietal region around 400 ms, consistent with past published descriptions of the N400 ERP.

**Fig. 6:**
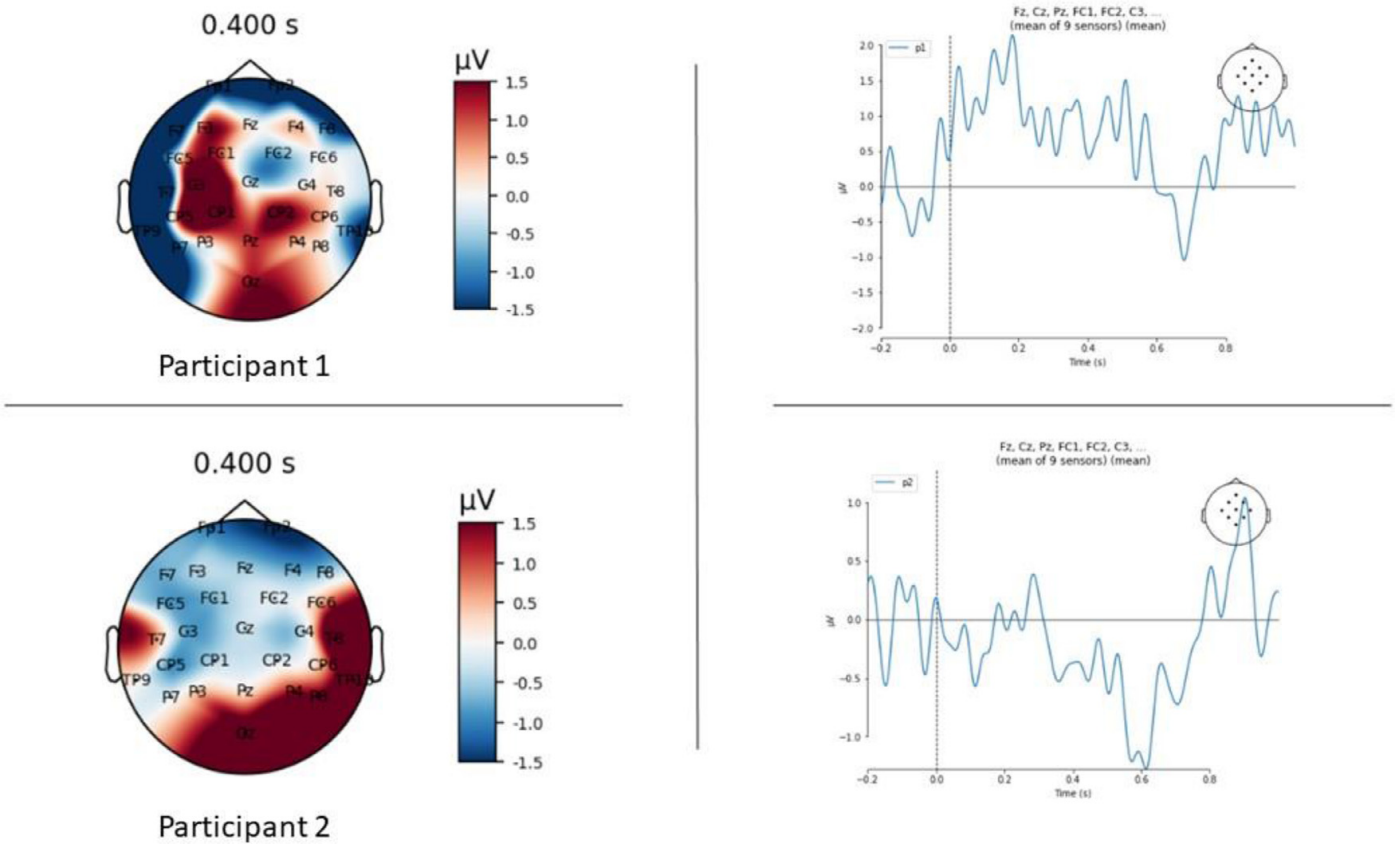
Data from two participant participating in a scripted conversation (Participant 1- above; Participant 2- below), averaged across all nouns heard by each participant. The left panels show scalp topography maps of activity averaged over 300–500 ms. The right panels show waveform plots of the average across a group of central-parietal electrodes (shown in the top right inset), time-locked to word onset (time 0). Participant 2 displays a negative potential over the midline central-parietal region around 400 ms, consistent with past published descriptions of the N400 ERP.

This is a subject-level demonstration of how to do ERP analyses after collecting hyperscanning data from a conversation, not the results of a full group-level analysis. It can be replicated using the step 1 and step 2 scripts provided alongside our supplementary data. For the datasets we have provided, the goal was to demonstrate that a hyperscanning method could be used for ERP experiments and that this technique allows researchers to precisely time-lock to words produced during a conversation. We have included two datasets, one from our free conversation experiment and the other from our scripted conversation paradigm. For example, we could use this method is to identify the N400 component that is typically elicited by open-class “content” words, such as nouns, in spoken and written language [18]. The N400 is characterized as a negative-going potential typically between 300 and 500 ms after word onset, largest over the vertex of the head relative to an averaged or averaged mastoid reference [18]. To demonstrate how to segment and process the data collected using our hyperscanning methods for an ERP analysis, we examined the data from individual participants  during the time window when the N400 effect would typically be observed. In Fig. 5, the response observed to content nouns from the free conversation experiment data is consistent with the predicted N400 as there is a negative potential around the central-parietal electrodes in the expected time range. We only show the free conversation data of participant 1 as there was considerable noise in participant 2′s data, however the data from both participants, including visualizations of the ERPs, are included in the supplementary material. In Fig. 6, we display plots of a waveform averaged over all trials of content nouns that occurred over the course of a scripted conversation that were heard by each participant in the dyad, as well as topographic maps averaged over 300–500 ms. Participant 2 in the scripted conversation experiment displays a negative potential around the central parietal region in the 400 ms time window in line with the N400 literature, whereas participant 1 does not. In terms of interpreting these responses- ERP analyses are group averages that compare responses to at least two conditions and the experiments typically contain at least a hundred stimuli, and it is well established that not every participant displays a typical ERP response [2].We provided two examples of negative potentials in individual participants occurring in the 400 ms time window in response to content words that occurred in conversational settings and that were detected in the central parietal electrodes, which is in line with a response that could contribute to an N400 ERP effect. However, purpose of the demonstration is to depict how this hyperscanning method can be used at the subject level to precisely time-lock to words that occur during a conversation for the purposes of an ERP experiment, not to perform a full group-level ERP analysis.

The results we present in this paper are from an initial pilot experiment that led to the design an execution of two experiments, each involving 40 participants. In those experiments we used a scripted conversation, rather than free conversation, to provide more consistency in the number and identity of content words for which ERPs were derived. Preliminary results of these have been reported  [19,20], and a manuscript is currently in preparation, but in short these experiments yielded robust N400 effects consistent with our demonstration here, and past single-participant research. Furthermore, Fjaellingsdal and colleagues recently [21] demonstrated that ERP responses (including the N400 ERP) occurred in participants equipped with a non-hyperscanning EEG setup who engaged in a spontaneous word-by-word sentence construction task with a non-EEG equipped confederate. A similar study could easily be conducted using the methods outlined in this paper where both people involved undergo EEG and are participants to examine if these findings hold out in slightly less controlled circumstances. Another single participant study examined the differences between how adults and infants processed the same mother-infant interaction [22]. There were ERP differences in processing contingent and non-contingent speech when listening to a 20-minute recording of a naturalistic free conversational interaction of a mother and her baby between the infants and adults. This indicates that there may be differences in cognitive processing across the life span when viewing the same interaction as infants displayed different ERP responses in comparison to the adult control group. Using the methodology outlined here, with only minor adaptations, would allow the examination and recording of infants and parent pairings while they themselves are engaged in conversational interactions, rather than being limited to examining only the outside observer role responses to these interactions.

### Conclusion

We developed methods and procedures to record EEG and audio simultaneously from a pair of participants using a single 64-electrode system, that allows us to conduct ERP experiment and analyses using stimuli that are words produced during a naturalistic conversation. This method could potentially be extended to recording from up to four people at once (using 16 electrodes each), and to a larger number of participants and/or number of electrodes per participant using an amplifier with more channels. The ability to perform hyperscanning using a single EEG amplifier provides a cost-effective approach that may make this technique available to a larger number of laboratories than previously possible. As well, our demonstration of how to record audio time-locked to the EEG data enables a range of ERP studies — such as conversation — that would otherwise be challenging to conduct in a hyperscanning study.

## Declaration of Competing Interest

The authors declare that they have no known competing financial interests or personal relationships that could have appeared to influence the work reported in this paper.

## Data Availability

We have provided links to our data.

## Appendix Supplementary materials

Image, application 1
