# Supplementary material for: A two for one special: EEG hyperscanning using a single-person EEG recording setup
Source: MethodsX. 2023 Feb 3;10:102019. doi: 10.1016/j.mex.2023.102019 (PMC9945774; doi:10.1016/j.mex.2023.102019)
Supplement: Supplementary file 1 [file mmc1.docx]

**Supplementary material *and/or* Additional information:**

The coding scripts and dataset mentioned in this paper have been provided alongside this article.
